# Ocular Involvement in a Pediatric Patient with Geleophysic Dysplasia

**DOI:** 10.3390/diagnostics16020193

**Published:** 2026-01-07

**Authors:** Bogumiła Wójcik-Niklewska, Zofia Oliwa, Paulina Sawuła, Adrian Smędowski

**Affiliations:** 1 Department of Pediatric Ophthalmology, Faculty of Medical Sciences in Katowice, Medical University of Silesia, 40-514 Katowice, Poland; bogumila_wojcik_niklewska@op.pl (B.W.-N.); asmedowski@sum.edu.pl (A.S.); 2 Professor Kornel Gibiński University Hospital Center, Medical University of Silesia, 40-514 Katowice, Poland; 3 Students’ Scientific Society, Department of Ophthalmology, Faculty of Medical Sciences in Katowice, Medical University of Silesia, 40-752 Katowice, Poland; s88051@365.sum.edu.pl; 4 GlaucoTech Co., 40-282 Katowice, Poland

**Keywords:** geleophysic dysplasia, FBN1 mutation, optic disc abnormalities, pediatric ophthalmology

## Abstract

Geleophysic dysplasia (GD) is a rare genetic skeletal disorder belonging to the acromelic group, characterized by short stature, distinctive facial features, thickened skin, and progressive cardiac involvement. We report a case of a 3-year-old boy with GD caused by a heterozygous c.5198G>A variant in the FBN1 gene, presenting with ocular abnormalities. The patient demonstrated coarse facial features, short hands and feet, and a history of mitral valve stenosis requiring mechanical valve replacement. He was referred to the ophthalmology department for evaluation of left eye strabismus and elevated intraocular pressure. Fundus examination revealed a pink optic disc with blurred margins, slightly elevated above the retinal plane, absent foveal reflex, and tortuous vessels, consistent with optic disc drusen on ocular ultrasonography. Photopic negative response (PhNR) testing showed markedly reduced amplitudes in both eyes, indicating retinal ganglion cell dysfunction. Pattern VEP revealed normal P100 latencies in both eyes, with a 30% reduction in amplitude in the left eye, likely related to poorer fixation. This case highlights optic disc drusen and retinal ganglion cell dysfunction as potential ocular manifestations of geleophysic dysplasia, emphasizing the need for comprehensive ophthalmologic evaluation in affected patients.

**Figure 1:**
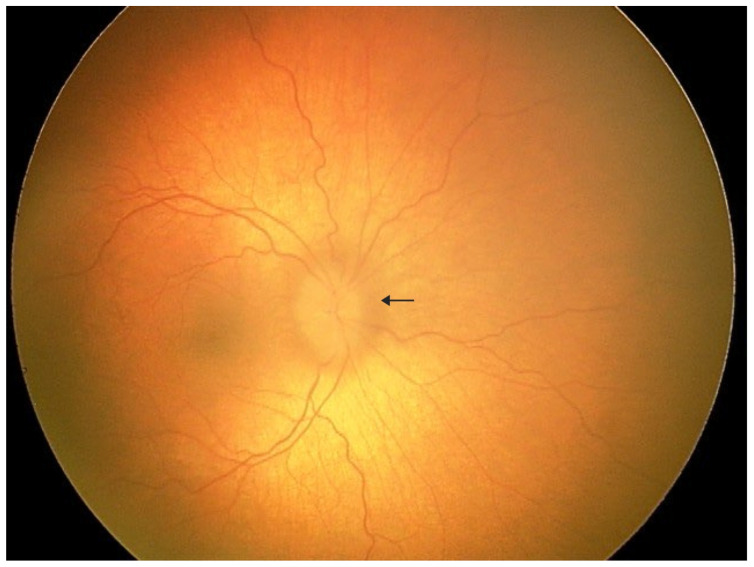
A 3-year-old boy was admitted to the pediatric ophthalmology department for diagnostic evaluation. The patient has a confirmed diagnosis of geleophysic dysplasia. He was born from the first pregnancy and first delivery at 40 weeks of gestation via spontaneous vaginal delivery, with a birth weight of 3640 g, length of 50 cm, head circumference of 34 cm, and Apgar score of 10. No congenital malformations were noted at birth; however, positional asymmetry was observed, and physiotherapy was initiated. At 5 months of age, failure to thrive and poor linear growth were noted. Despite nutritional interventions under gastroenterological supervision, growth parameters remained below normal. Abdominal ultrasound revealed hepatomegaly, and echocardiography demonstrated mitral valve stenosis, which required mechanical valve implantation. Physical examination showed coarse facial features, reversed epicanthal folds, broad nasal bridge, mild brachydactyly, short and broad hands, and broad feet. Mucopolysaccharidosis was excluded. Genetic testing revealed a heterozygous c.5198G>A variant in the FBN1 gene, not present in either parent. Based on the clinical presentation and genetic findings, geleophysic dysplasia was diagnosed. Initial ophthalmologic examination showed hyperopia of +5.0 D and astigmatism. The patient was referred from the emergency department to the ophthalmology ward for further evaluation due to elevated intraocular pressure (IOP) in the left eye, blurred optic disc margins, and left convergent strabismus. Under general anesthesia, detailed ophthalmologic assessment was performed. IOP measured 19 mmHg in the right eye and 22 mmHg in the left eye. Although the IOP in the left eye was slightly above the upper pediatric reference range, it did not meet diagnostic criteria for ocular hypertension or glaucoma, as no structural or functional glaucomatous changes were present. Refractometry demonstrated hyperopia of +3.75 D in the right eye and +5.75 D in the left eye with significant astigmatism in both eyes. Fundus examination of the left eye revealed a pink optic disc with blurred margins, slightly elevated above the retinal plane, absent foveal reflex, and tortuous vessels. The arrow indicates the blurred optic disc margin.

**Figure 2:**
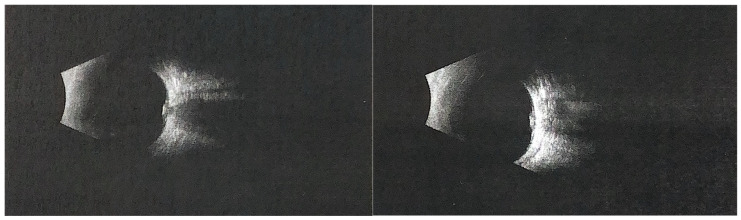
The (**left**) panel represents the right eye, and the (**right**) panel represents the left eye. Ocular ultrasonography of the left eye showed optic disc border enlargement and an area of increased echogenicity, suggesting superficial optic disc drusen. Photopic negative response (PhNR) recordings demonstrated markedly reduced amplitudes and W-ratio values in both eyes, indicating dysfunction of retinal ganglion cells. Pattern VEP testing revealed normal P100 latencies in both eyes, while the P100 amplitude in the left eye was approximately 30% lower than in the right eye, possibly influenced by fixation instability. Geleophysic dysplasia (GD) is a rare skeletal dysplasia within the acromelic group, which also includes acromicric dysplasia (AD) and Weill–Marchesani syndrome (WMS). These disorders share overlapping characteristics such as short stature, short hands and feet, progressive joint limitations and contractures, skin thickening, distinctive facial features, and abnormal skeletal morphology [1,2,3]. In contrast to AD, which typically has a milder outcome due to the absence of progressive valvular disease, GD is associated with severe, progressive cardiac involvement that often determines prognosis [1,2]. GD is characterized by proportionate short stature, prominent abnormalities in the hands, feet, bones, and joints, and progressive cardiac, respiratory, and pulmonary pathology, combined with a characteristic facial appearance (from the Greek gelios = happy, physis = nature) [1,2,3]. The condition is most commonly caused by autosomal dominant mutations in FBN1 (geleophysic dysplasia 2), while autosomal recessive cases due to ADAMTSL2 mutations (geleophysic dysplasia 1) have also been described [1,2]. The FBN1 c.5198G>A variant identified in our patient has previously been reported in a child with geleophysic dysplasia, presenting with severe pulmonary hypertension and hepatomegaly, but without available information on ocular manifestations [4]. Our case therefore expands the phenotypic description of this variant by documenting optic disc drusen and pseudopapilledema in this clinical context. Mutations in FBN1 are associated with a wide spectrum of connective tissue disorders, including Marfan syndrome and Weill–Marchesani syndrome, although the mechanisms leading to both tall and short stature remain incompletely understood [2]. Fibrillin-1, encoded by FBN1, is a major component of extracellular microfibrils and elastic fibers, essential for the structural integrity and signaling functions of the extracellular matrix [5]. Disruption of fibrillin-1 may alter microfibril organization and the mechanical stability of connective tissues, including the peripapillary region of the optic nerve head. In this setting, disruption of axonal transport could favor the accumulation of deposits within the optic nerve head and contribute to optic disc drusen formation [6]. Although a direct causal link has not been demonstrated, our findings suggest that FBN1-related microfibril dysfunction might contribute to the development of pseudopapilledema in these patients. The true prevalence of GD is unknown; however, fewer than one hundred cases have been reported worldwide, and many affected individuals may remain undiagnosed or misdiagnosed due to the variability of clinical presentation [1]. Ocular involvement in GD, although less frequent than systemic features, is clinically significant and increasingly recognized. Reported findings include optic disc swelling, which may mimic papilledema and pose diagnostic challenges [7]. In our patient, the blurred and slightly elevated optic disc initially raised concern for papilledema. However, B-scan ultrasonography revealed highly echogenic deposits consistent with optic disc drusen, confirming pseudopapilledema. Since papilledema implies possible intracranial hypertension, this diagnosis was considered; however, the child had no neurological symptoms or clinical signs suggestive of raised intracranial pressure. Therefore, further neuroimaging was not pursued. Additional manifestations may include refractive errors, strabismus, corneal clouding, or glaucoma [8,9]. Bilateral angle-closure glaucoma has also been reported in association with GD and may represent a vision-threatening complication if not promptly managed [9]. Importantly, ocular abnormalities can present early in the disease course, sometimes serving as a valuable diagnostic clue for clinicians [8]. For this reason, comprehensive ophthalmological evaluation should be considered an essential component of the multidisciplinary management of GD.

## Data Availability

No new data were created or analyzed in this study. Data sharing is not applicable to this article.
